# Inference of Causal Networks from Time-Varying Transcriptome Data via Sparse Coding

**DOI:** 10.1371/journal.pone.0042306

**Published:** 2012-08-20

**Authors:** Kai Zhang, Ju Han, Torsten Groesser, Gerald Fontenay, Bahram Parvin

**Affiliations:** Life Sciences Division, Lawrence Berkeley National Laboratory, Berkeley, California, United States of America; University of California, Davis, United States of America

## Abstract

Temporal analysis of genome-wide data can provide insights into the underlying mechanism of the biological processes in two ways. First, grouping the temporal data provides a richer, more robust representation of the underlying processes that are co-regulated. The net result is a significant dimensional reduction of the genome-wide array data into a smaller set of vocabularies for bioinformatics analysis. Second, the computed set of time-course vocabularies can be interrogated for a potential causal network that can shed light on the underlying interactions. The method is coupled with an experiment for investigating responses to high doses of ionizing radiation with and without a small priming dose. From a computational perspective, inference of a causal network can rapidly become computationally intractable with the increasing number of variables. Additionally, from a bioinformatics perspective, larger networks always hinder interpretation. Therefore, our method focuses on inferring the simplest network that is computationally tractable and interpretable. The method first reduces the number of temporal variables through consensus clustering to reveal a small set of temporal templates. It then enforces simplicity in the network configuration through the sparsity constraint, which is further regularized by requiring continuity between consecutive time points. We present intermediate results for each computational step, and apply our method to a time-course transcriptome dataset for a cell line receiving a challenge dose of ionizing radiation with and without a prior priming dose. Our analyses indicate that (i) the priming dose increases the diversity of the computed templates (e.g., diversity of transcriptome signatures); thus, increasing the network complexity; (ii) as a result of the priming dose, there are a number of unique templates with delayed and oscillatory profiles; and (iii) radiation-induced stress responses are enriched through pathway and subnetwork studies.

## Introduction

Biological systems often operate as networks of interacting components that are highly regulated [1]. These networks enable a cell to integrate external stimuli and biochemical reactions that can potentially lead to the activation of transcription factors (TFs). In turn, these TFs recognize a specific regulatory region for manipulating gene expressions. Characterization of network biology has been further advanced through mathematical analysis of genome-wide array data for hypothesis generation. In the context of mathematical modeling, logical (e.g., Boolean [2], stochastic [3], [4], petri net [5]) and continuous (e.g., ordinary differential equations [6], flux balance analysis [7]) techniques have been proposed. Recent reviews of these techniques can be found in [8], [9]. Each of these techniques has its own pros and cons with distinct application domains. In this paper, we introduce a method to hypothesize a causal network that is derived from the analysis of the time-varying genome-wide array data, where causality is interpreted in a weak sense to show a potential relationship between groups of transcripts at two consecutive time-points. Given the complexities of a biological network and inherently high dimensionality of an array-based data coupled with a low sample size, we aim at deriving the simplest network for hypothesizing causality. We suggest that causality can be inferred through either perturbation studies or time-course data. The latter has the potential to enrich the genome-wide array data by grouping time-course profiles; thereby, leading to a lower dimensional representation. Subsequently, such a low dimensional representation can then be modeled as a layered signaling network, where each output at a given time layer is expressed as a function of inputs from a previous time point. The net result is a causal network (e.g., a wiring diagram) that fits the time-varying data according to a cost function. The concept of “simplicity” is enforced by requiring that (i) not all input variables from a given time point contribute to an output at the next time point, (ii) an output is a linear combination of input variables, and (iii) there is a notion of continuity in the signaling network. Collectively, these constraints lead to a highly regularized sparse linear model. The method is validated against different configurations of synthetic data and then applied to an experimental dataset to examine the effects of a higher dose of ionizing radiation with and without a priming low dose of radiation, which is known as an adaptive response. The proposed computational protocol is applied to a unique experiment in radiation biology, where a cell line has been treated in one of two different ways: (a) with a challenge dose of 200 cGy or (b) with a priming dose of 10 cGy followed by the challenge dose of 200 cGy. The latter is referred to as an “adaptive response” [10]–[12], since adaptation is attributed to reduced damages as a result of adding the priming dose. Consequently, it is our goal to characterize and differentiate induced perturbations in terms of the (a) shape and number of computed templates, (b) architecture of the wiring diagrams, and (c) biological interpretation through enrichment analysis.

## Results

We provide an analysis of clustered temporal profiles, followed by an interpretation of the causal networks.

### Analysis of temporal profiles

**Figure 1:**
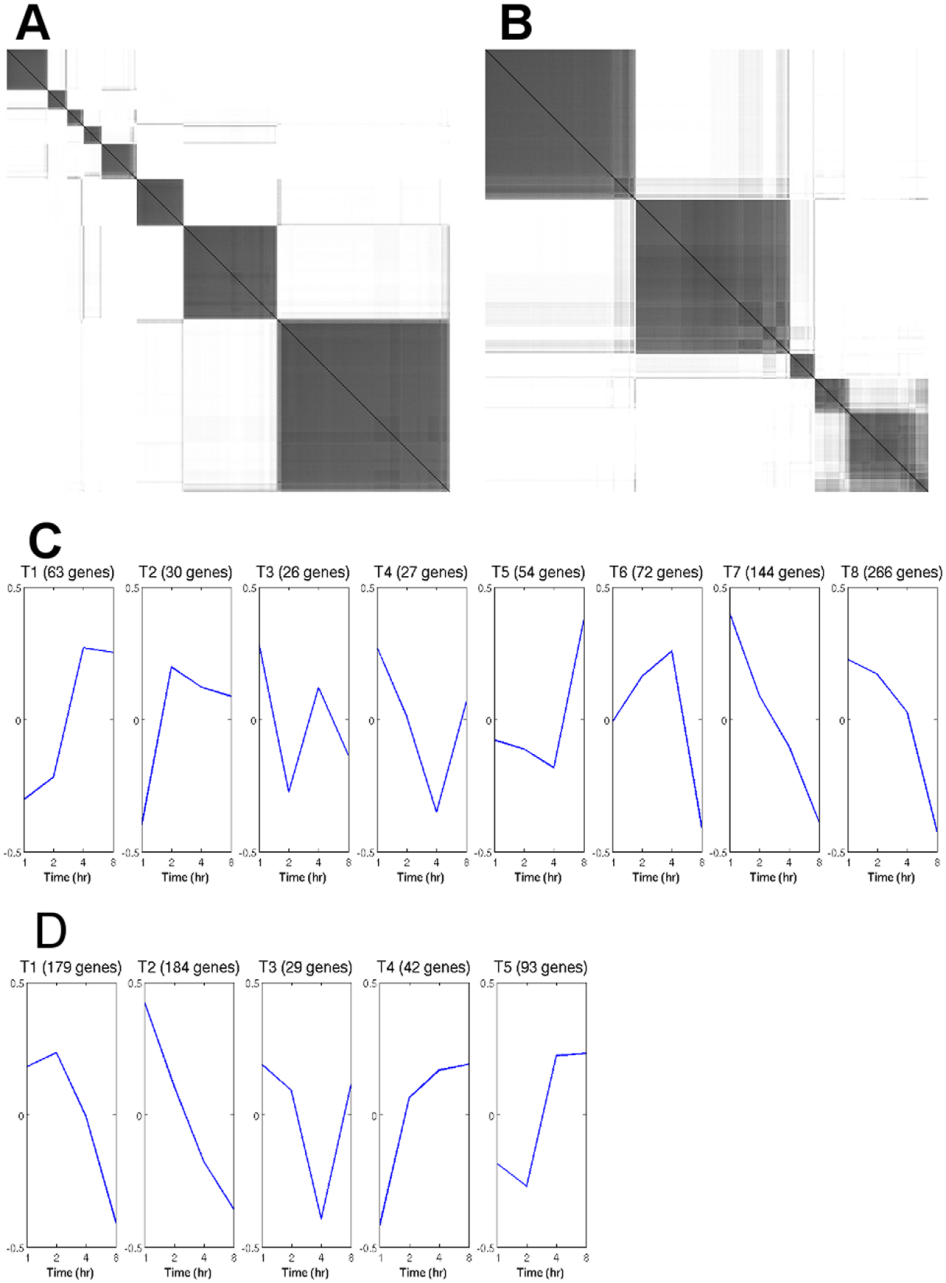
Analysis of temporal profile. Consensus clustering indicates A) 8 clusters for transcript data with the priming dose and B) 5 clusters in the control group. Each cluster, in A) and B), corresponds to a unique temporal profile as shown in C) and D), respectively.

**Figure 2:**
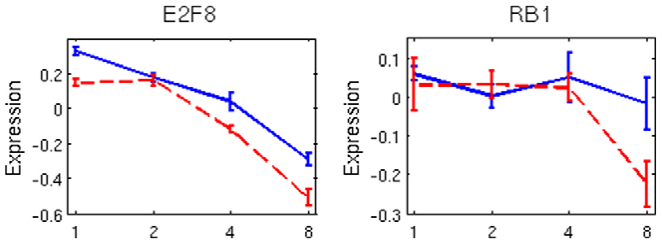
Transcriptome profile of E2F family and RB1 indicates down-regulation for the treatment group with the priming dose. RB1 remains unchanged in the absence of the priming dose. Dash red-lines correspond to adaptive response.

The initial sets of gene expression data for the treatment groups with and without the priming dose are reduced to 682 and 527 genes, respectively, in accordance with the policy outlined in the Method section. These genes have highly variable expression values across different time points. Consensus clustering of filtered transcript data indicates there are 8 clusters that correspond to samples receiving the priming dose (e.g., adaptive response) versus the 5 clusters that do not receive the priming dose (e.g., challenge), as shown in Figures 1A and 1B. Each cluster from each of the treatment groups corresponds to a unique temporal profile as shown in Figures 2C and 2D. The details for selecting the number of clusters are summarized in Figures S1, S2, S3, S4, and Text S1. We label each template, in its own cluster, as 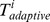 and 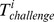, where *i* represents the template number (e.g., 1–8 for those *with* the priming dose), *adaptive* represents those samples receiving the priming dose prior to the challenge dose, and *challenge* representing those that only receive the challenge dose.

A comparison of Figures 1C and 1D indicate both similarities and dissimilarities in the radiation response with and without the priming dose. These differences can then be used to probe for bioinformatics analysis. We used *Pathway Studio* to analyze the computed clusters through pathway and subnetwork enrichment analysis for identical and differential responses with the results shown in Tables 1 and 2.

**Table 1:** Ariadne signaling pathway and subnetwork enrichment with priming dose with the default p-value of 0.05.

Templates with priming dose	Signaling pathway enrichment	Subnetwork enrichment
T1	Apoptosis regulation, TNFR->(AP1, NF-kB, CREB), IL1R->STAT3, FOXO3A with p-value<0.02	TP53, SP1, GF, TNF, IL1B, TGFB1, IFNG, Cytokines with p-value<0.004
T2	TP53 signaling with p-value<0.008	TP53, TP73, STAT1, E2F1, interferon, ESR1, CREB1, NF-kB, IFNG, SP1
T3	(TNFR,TLR3, CSF2R, DDR1)->NF-kB, Mast cell activation, DDIT3, HSF1, AP-1 with p-value<0.03	NF-kB, TGFB1, TNF
T4	(HGFR, PDGFR, VEGFR)->FOX03A, multiple receptors->NF-kB signaling	Proteasome endopeptidase complex with p-value<0.03
T5	STAT signaling	POU2F1, CEBPA, RCA1
T6	Hedgehog, Cell cycle, Notch,STAT3, STAT, STAT1	NOG, PDGF, EDN1, IL1A
T7	Cell cycle regulation with p-value<0.0002	IGF1, VEGFA, Ras
T8	Cell Cycle regulation, hedgehog	E2F, RB1, TP53, MYC, PDGF

**Table 2:** Pathway and subnetwork enrichment without the priming dose with the default p-value of 0.05.

Templates without the priming dose	Signaling pathway enrichment	Subnetwork enrichment
T1	Hedgehog, Cell cycle regulation	E2F, TP53, RB1, MYC, SP1, PDGF
T2	Cell cycle, Notch->(MEF, TCF3, NF-kB)	Proteasome endopeptidase complex, TP53, cytolines, NF-kB, IL1B, TGFB1, TNF with p-value<0.01
T3	none	EGR1, GH1
T4	TNFR->(AP1, TP53, NF-kB, CREB), CD43, TNFRSF6->(HSF1, FOXO3A, RB1, E2F)	TP53, AKT1, MAP, SP1, NF-kB, IFANG, TNF with p-value<0.02
T5	Cell cycle regulation, Apoptosis	TP53, SP1, IL1B, EGF, TNF

Similar profiles such as: (i) 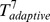 and 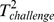 appear to have a down-regulated profile from 1 to 8 hours. Here, the pathway and subnetwork enrichment analysis reveals a significant amount of overlap through the cell cycle regulation and inflammatory responses. (ii) 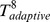 and 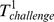 are initially flat and then down-regulated with similar pathway (e.g., cell cycle and Hedgehog) and subnetwork (e.g., E2F, RB1, TP53, PDGF) enrichment analyses. The role of E2F and RB1 in regulating cell cycle, cell fate, DNA damage repair and apoptosis has been well established [13], [14]. It has also been shown that the cellular response to DNA damage utilizes the RB/E2F cell cycle pathway [15]. Their time profiles are shown in Figure 2, where RB1 is significantly down-regulated in samples receiving the priming dose. The E2F family shows similar profile for its transcripts; a representative, E2F8, is also shown here. (iii) Conversely, 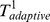 and 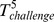 share the same temporal signature, but in the opposite direction of the templates in (ii). Specifically, they are initially flat, but then upregulated. These templates share a significant amount of overlap in terms of pathway (e.g., apoptosis) and subnetwork (e.g., TP53, SP1, IL1B, and EGFR) enrichment analyses. (iv) Although 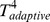 and 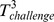 also has similar temporal profiles, the ensemble of genes representing 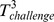 are poorly enriched, which is largely due to the lack of related annotation data. On the other hand, 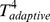 is highly enriched for FOXO3A and NF-kB. (v) Lastly, 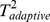 and 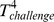 are initially upregulated and then plateau after the *2* hour time point with the enrichment of CD43 (for regulating immune function) and TP53 pathways in adaptive and challenged response treatment groups, respectively. These analyses suggest that every template in the challenge group has a profile similar to those in the treatment group with the priming dose, and in one case, pathway enrichment has been limited only to the immune function activation.

**Figure 3:**
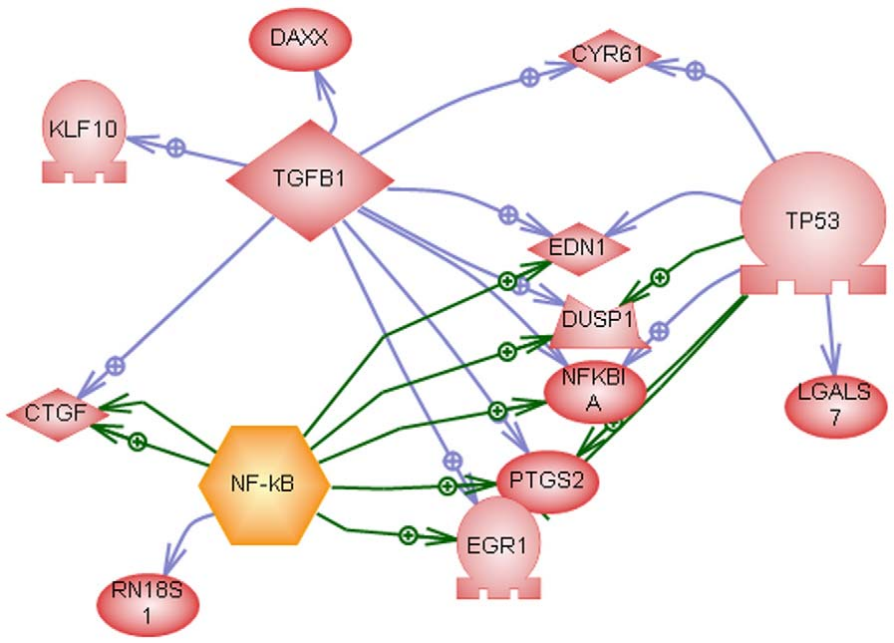
Subnetwork enrichment analysis of template 3 with priming dose.

**Figure 4:**
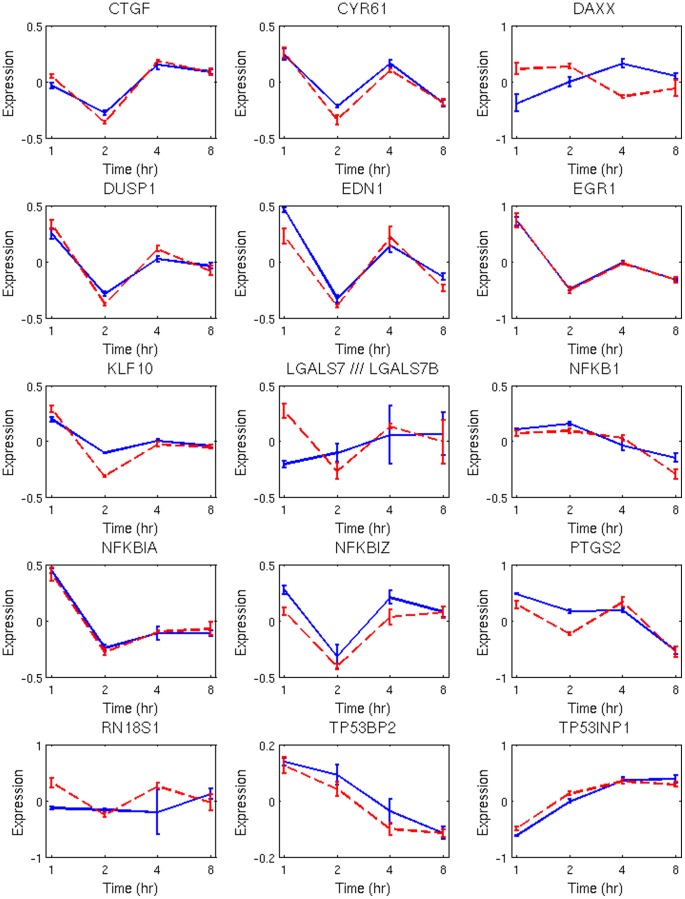
Temporal profiles of endpoints in **Figure 4** with a significant fold change. Dash red-lines correspond to adaptive response.

Dissimilar profiles are 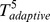 and 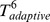 (upregulated and downregulated, respectively, at the *4* hour time point) as well as the oscillatory profile of 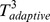. The first two templates indicate a delayed response as a result of the priming dose, and enrichment analysis indicates a number of components that overlap with the existing templates. In 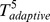 and 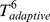, STAT signaling is an enriched dominant pathway that integrates cellular stresses such as ultraviolet radiation, inflammation, and infection [16], as reflected in the subnetwork enrichment analysis and reported earlier as a result of low dose radiation on inbred strains of mice [17]. Further examination indicates that 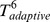 shares some of the components of 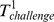, but with a delayed response, as shown in the supplementary section. These shared transcripts, for example, HIST1H4B, are nuclear-bound and are involved in chromatin remodeling. However, the subnetwork enrichment analysis of the oscillatory profile of 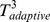 indicates enrichment of NF-kB, TP53, and TGFB1, shown in Figure 3. Figure 4 shows the temporal profiles for a subset of transcripts presented in Figure 3, which are also known to be sensitive to ionizing radiation. Although the oscillatory signature is more dominant in the adaptive response where it forms a distinct cluster (e.g., template), some of the transcripts in the challenge groups also show similar profiles. Finally, TGFB1 has been identified as a common regulator of radiation response in Figure 4, though its transcriptome profile is not significantly altered as a result of ionizing radiation.

### Analysis of causal network

**Figure 5:**
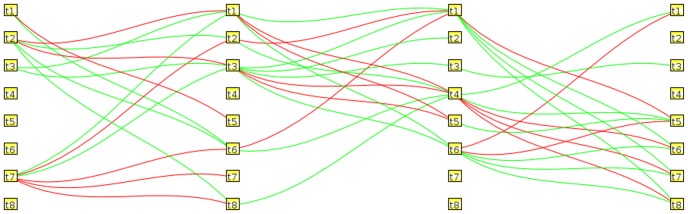
Causal networks for the 8 templates with priming dose in **Figure 2C**.

**Figure 6:**
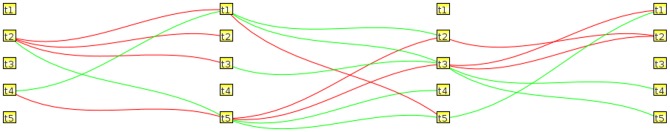
Causal networks for the 5 templates without priming dose in **Figure 2D**.

Causal networks, shown in Figures 5 and 6, indicate interactions between computed temporal profiles. These networks are constrained to infer the simplest wiring diagram, in terms of directed graphs, for interpretation. The wiring diagram provides the means for interpretation of each computed template and its role in the dynamical system. Below, we analyze both the initial and final active templates.

The initial active templates are 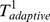, 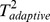, 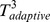, 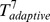, 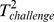 and 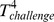, and bioinformatic analyses suggest that the network is enriched by cell cycle, inflammatory, and apoptosis regulation processes. Although for the treatment group receiving the priming dose, enrichment is highly biased with inflammatory and immune responses. It is also clear that the oscillatory template 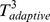 and delayed activation 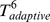 play a role in the adaptive response (e.g., the treatment group receiving the priming dose) at an intermediate stage. Had there been no connections, then these templates would have been non-essential.

**Figure 7:**
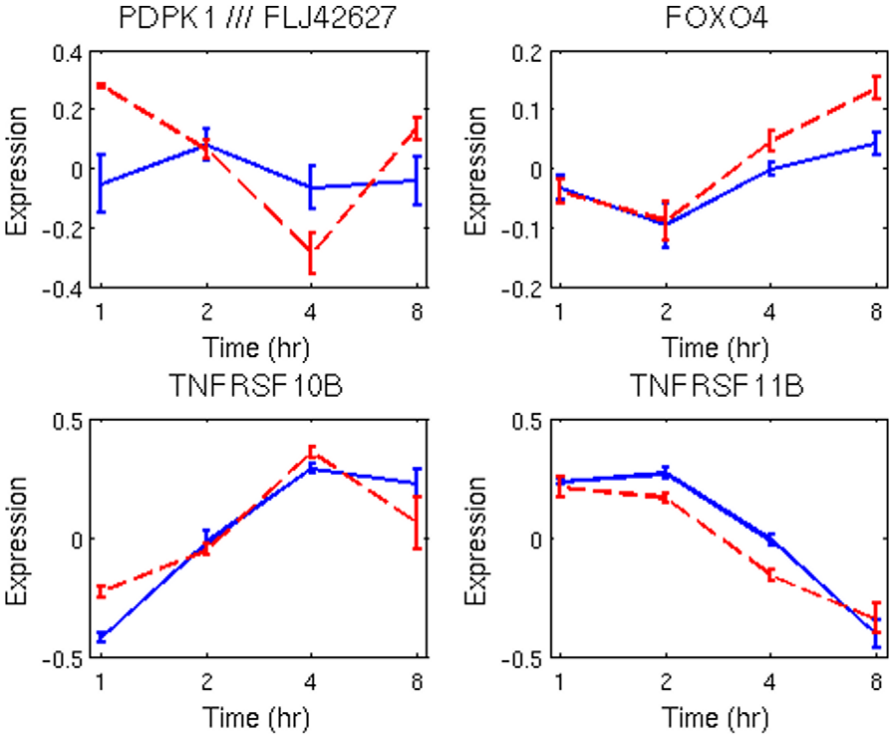
Temporal profiles of PDPK1, FOXO4, and TNF family involved in activation and inhibition of NFkB. Dash red-lines correspond to adaptive response.

The final active templates (e.g., the interval between 4–8 hour time point) are largely enriched by the identical temporal signatures in 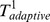, 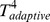, 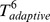, and 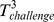. While FOXO3A and NFkB pathways are highly enriched through multiple receptors with the group receiving the priming dose, enrichment in the challenge group is limited to EGR1. Enrichment of FOXO3A is potentially due to PDPK1, which is differentially expressed between the two treatment groups, as shown in the top row of Figure 7. However, there is no differentially expressed signal in FOXO3A transcription factor even though EGR1 and FOXO3A are shown to be associated with the exposure to the ionizing radiation [18], [19]. EGR1 functions as a hub for many signaling cascades that is essential for growth and proliferation [20], and is down-regulated as a result of ionizing radiation in both groups. Within the FOXO group, FOXO4 has a slight upregulation in both treatment groups. FOXO transcription factors are highly conserved genetic pathways, at the intersection of aging and cancer, that are phosphorylated in response to insulin and growth factors [21]. Upregulation of FOXO can cause apoptosis through an independent p53 pathway [19], whereas loss of Forkhead FOXO transcription factors in a cancer cell may decrease cell cycle arrest or apoptosis as a result of DNA damage or genomic instability [22], [23]. Enrichment of NFkB is potentially through the TNF family. More specifically, TNFRSF10B is an activator of NFkB while TNFRSF11B is an inhibitor of NFkB. Their profiles are shown again in Figure 7 (lower row).

## Discussion

From the perspective of a strict gene expression, the fold changes are generally low and appear to be stochastic as a result of ionizing radiation. This observation is consistent with previous literature [24], [25]. Nevertheless, the temporal patterns from the gene expression provide more candidates and are more informative than a single time point observation, i.e., any transcript with a small value, at a given time point, can be eliminated using standard filtering techniques. The richness of the temporal gene expressions is crucial in grouping and hypothesizing causal relationships from high dimensional transcriptome data. Typically, inference of the causal relationships can be ambiguous; there is significant literature in support of it [26] and against it [27], but most researchers agree that through carefully designed experimental data, ambiguities in the inference of causation can be reduced or eliminated. Such an experimental design may include a specific set of perturbations (e.g., siRNA) that may also include the time-course data. The time-course enables identification of a set of similar profiles that will (i) reduce complexities in the causal network, (ii) provide pseudo replicates for sampling and cross validation, and (iii) constrain the network structure (and the solution) by enforcing temporal continuity. In short, the proposed computational protocol enables interpretation of a complex dataset at multiple steps. However, the main theme is inference of the simplest network that is computationally tractable, and at the same time, interpretable. The method is initiated by identifying temporally co-regulated transcripts into a distinct set of templates or groups. This step not only reduces the dimensionality of the data, but also reduces the number of variables that need to be estimated for building the causal network, i.e., transition matrices. The network construction assumes a model for which every node, at a given time point, is a sparse linear combination of nodes in the previous time point. The concept of sparseness also enforces the notion of network simplicity. Finally, the solution is regularized by eliciting continuity of the transition matrices between consecutive time points. It should be noted the method has been applied to transcriptome data, but it is also extensible to other time-course data, i.e., identifying aberrant signal transduction pathways.

The method has been validated on synthetic data and then applied to transcriptome data that has been collected from a cell strain, which was exposed to 2 Gy ionizing radiation (e.g., the challenge dose) with and without the priming dose of 10 cGy applied 4 hours prior to the higher dose of radiation. Bioinformatics analyses revealed that computed templates (e.g., clusters) without the priming dose (e.g., in the challenge group) are a subset of those that received the priming dose (e.g., the adaptive response group). Furthermore, the adaptive response group included templates with delayed activations and oscillatory behavior. It is clear that the priming dose has induced a significant amount of diversity in how the networks are modulated. In both treatment groups, the initial active templates of the causal networks are highly enriched by the down-regulation of the cell cycle machinery. However, in the case of the adaptive causal network, the network is also modulated by the up-regulation of the inflammatory processes. On the other hand, with the exception of EGR-1, the network is poorly enriched at the late stages for the treatment group that does not receive a priming dose. It has been suggested that both EGR-1 and p53 are essential for mediating radiation-induced apoptosis [28]. The effect of priming at a late stage indicates a network modulation through pro-inflammatory responses and proteasomes, which is also consistent with the literature on low dose exposure [29], [30].

Another way to examine experimental data is through enrichment of cellular processes. Initially, both treatment groups are enriched by DNA double strand repair, apoptosis, and cell cycle processes. However, the group receiving the priming dose is also enriched with single strand base excision and mismatch DNA repair. Within the group receiving the priming dose, these processes are modulated with a chromatin remodeling (e.g., 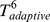) at 8 hour time point. On the other hand, the group receiving no priming dose appears to be poorly enriched in the final stages. The bioinformatics analysis suggests that the priming dose changes the network architecture by delaying the effects of the chromatin remodeling.

## Materials and Methods

### Experimental design

The experiment is designed around the human diploid, embryonic lung, fibroblast cell strain WI-38 (TP53 proficient) [31], [32], which is publicly available from the Coriell Institute. These cells were grown as a monolayer (2D) under a physiologically relevant oxygen concentration (3%) with 10% CO2, instead of at high oxygen levels (about 20%). Additionally, the cells were asynchronously growing when exposed to 2 Gy (e.g., the challenge dose) of ionizing radiation (160 kV X-rays), with or without a priming dose of 10 cGy (e.g., the adaptive dose), 4 hours prior to the challenge dose. Three biological replicates for each treatment group (e.g., with and without the priming dose) were collected 1, 2, 4 and 8 hours after the challenge dose. The time-course was selected on the basis of our prior research on early responses to ionizing radiation [33], [34]. Purified total cellular RNA was extracted using an RNeasy Mini Kit (Qiagen) and quantified for Affymetrix microarray analysis using the Human Gene 1.0 ST Array. A robust multi-array analysis (RMA) was performed to the normalize data collected from the different samples. Samples were then examined for quality control using the NUSE protocol, which is shown in Figure S5 and Text S2. The array data is publicly available with accession number of GSE37688.

### Computing temporal templates from gene expression data

The first step of our protocol is to eliminate transcripts with little variation, which are maximum folds of change less than *0.5*. The net result is a significant reduction in the number of candidate transcripts, with those having similar temporal profiles being grouped together. The basic assumption is that co-regulated transcripts have a similar biological basis and is a step towards significant dimensionality reduction through clustering and categorization. Currently, there is an abundance of literature available on the clustering of time-varying expression data that includes predefined templates [35], autoregressive models [36], curve-based clustering [37], and mixture models [38]. Nonetheless, this is not the main theme of our research. Our approach relies on constructing template profiles through consensus clustering [39], widely used for class discovery, and then leveraging higher level enrichment analysis for evaluation. Consensus uses a voting strategy on the resampled data, from different runs, with a clustering algorithm (e.g., *k*-means), and facilitates visualizing of computed clusters for quality control. In our implementation, the clustering algorithm is based on *k*-means, where the distance measured is one minus the sample correlation. The clustering procedure is repeated for 1000 runs, and each run is performed on the randomly sampled genes with a sampling rate of 0.8. The optimal number of clusters is determined by examining the clustering stability and similarity matrix.

### Inference of causal networks

Suppose we have clustered genes into *K* groups, and each group contains 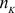 genes, i.e., 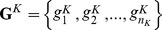. All the genes in the same group are assumed to have similar temporal patterns, which can be thought of as being denoted by a representative pattern, 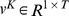, where *T* is the number of time frames. By concatenating the representative pattern for the *K* groups, we can obtain a *K*-by-*T* data matrix
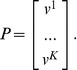
(1)Our objective is to analyze the temporal causal relationships among the gene expression profiles for the *K* groups, i.e., whether the expression level of group-*i* at time *t* will have an impact on the expression level of group-*j*. More systematically, we use the following matrix equation to encode the causal relationships,
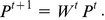
(2)Here, 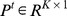 denotes the *t*-th column of *V*, and 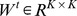 is the time-varying coefficient matrix, where 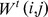 depicts how the expression level for group-*j* at time *t* affects the expression level of group-*i* at time *t+1*. A positive 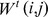
indicates a positive correlation, and a negative one means a depression in the expression level.

However, in practice, we are confronted with the big challenge of processing limited data while estimating the large parameter space of 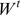. Additionally, more complications are introduced as a result of the time-varying nature of 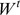
which increases the number of parameters by a factor of *T*. To alleviate the lack of samples in the inference problem, we propose the following extensions:

(i) A temporal regression framework for the time course data.

**Figure 8:**
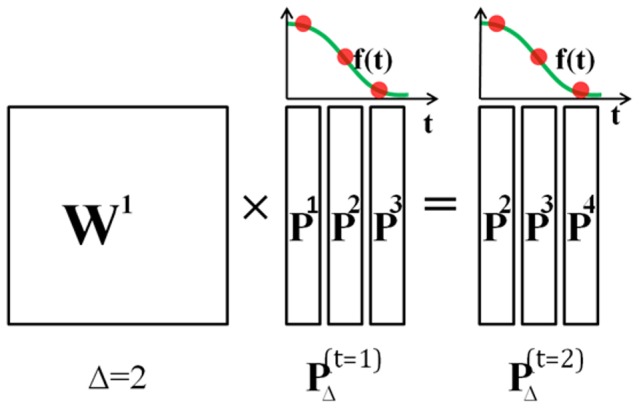
Illustration of the sliding window regression.

Instead of using only one column in Equation (2), we will apply a few columns together, corresponding to a small sliding window (multiple adjacent time frames) in the forward direction:
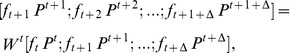
(3)Where 
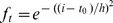
 is a weighting function that assigns smoothly decaying weight to the distance between *t* and the current centering frame 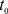. We simplify the notation of the linear equation group (3) by
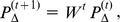
(4)where 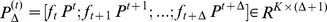 This is illustrated in Figure 8, where we use a sliding window of Δ = 2. As one can see, for current centering frame 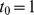, the equality 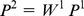 is assigned the largest weight, 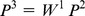 has a smaller weight, and 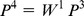 has a diminishing weight. This means that when computing 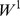, most of the emphasis should be placed on the constraint closest to the current time frame, i.e., the relationship between 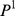 and 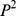. By doing this, we equivalently increase the number of samples used to compute each 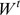, while at the same time assigning smaller weights to samples that are temporally too far away from the current centering frame. Mathematically, we use the regression framework for computing the coefficient matrices,
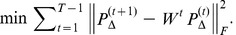
(5)


(ii) The coefficient matrices should vary smoothly with time.

Temporal coherence of 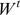 is an important constraint that can be utilized to enforce reliable estimations. We assume that the mechanism for gene-gene interactions is unlikely to change drastically over small time intervals. This is then translated into a smoothness term for 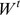
's. More explicitly, if *t1* and *t2* are in close proximity to each other (according to a predefined range), then 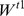 and 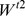 should also be close to each other. To enforce similarity between adjacent
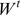
's, we also include the following term
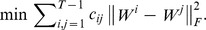
Here, 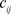 is an indicating factor that determines whether *i* and *j* are close to each other: if so, 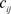 should be a positive number, enforcing the closeness between 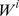 and 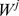 in the above minimization term; otherwise 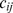 will be zero, indicating no constraints on 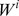 and 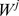 if *i* and *j* are far away. In practice, one can have either a hard indicator,
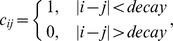
or a soft indicator,
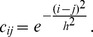



(iii) The coefficient matrices are sparse.

We note that the matrix 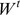 uniquely specifies a directed graph, where each node is a gene group and the edge weights indicate the interaction between the groups. However, only a subset of genes is active at any time point, which means that the network structure is sparse, i.e., only a small portion of the node pairs have interactions with each other. The notion of sparsity can be enforced via penalizing the 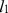-norm of the coefficient matrix, as
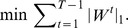
This is typically known as sparse constraint. In recent years, it has been applied extensively in signal processing, image reconstruction, and model selection [40], [41].

By combining the three terms, we have the following optimization problem
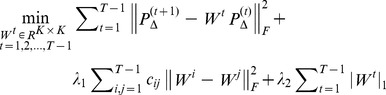
Here, 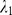 and 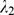 are positive regularization parameters that control the balance between the loss term, the temporal smoothness term, and the sparsity of solutions.

### Using multiple replicates for regression

As we previously indicated, a critical issue is the low sample size given the high dimensionality of the parameter space. Thus, to improve robustness and stability of the solution, we adopt the policy of using individual transcripts as replicates as they have similar signatures within a clustered group. In practice, one can compute a representative expression profile 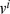 for the *i-*th group of genes, as discussed. However, in some cases, genes in the same cluster still have a certain level of variation, and using their average pattern for regression might lead to loss of information. To solve this problem and be able to fully utilize available patterns, we randomly sample genes from each group as the representative 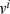 to form the data matrix (1); we then repeat this process and create an altogether *N* data matrices; each data matrix will lead to one objective term, as specified in (4). We will then sum up the objective terms associated with all the data matrices as the ultimate objective function. We can sample as many times as needed, i.e., injecting more constraints to the optimization problem, using certain heuristics for sampling. For example, given *K* groups and 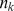 genes for *k*-th group, then the total number of different data matrices can be 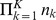.

### Optimization protocol

There are two ways to obtain the solution: (i) concatenate the columns of each 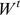 as a 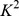-by-1 vector, and concatenate the *T-1* matrices to form a 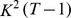-by-1 vector as the variable. The whole objective function can be transformed to a standard quadratic programming problem with a sparsity constraint, which can then be transformed into a 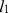-regularized least-square problem for which many advanced solvers are available; or (ii) the block coordinate descent method can be incorporated. Instead of computing all the variables at one time, we update one 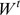 matrix at a time while fixing all other 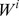
's (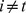). The first approach requires larger memory due to the need to manipulate the 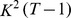-by-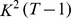 Hessian matrix. In practice, when *K* is very large, we apply the Nystrom low-rank approximation by sampling only a subset of the rows/columns of the Hessian matrix. This allows us to maintain enough energy in the eigen-spectrum in the reconstructed Hessian. The second approach is more memory efficient, but may require many cycling updates to converge.

### Selection of regularization parameters

The evaluation of regularization parameters 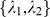 is based on Bayesian information criterion (BIC). Similar to Ahmed and Xing [42], we define the BIC as
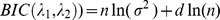
Where 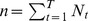, 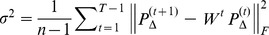, and 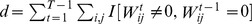. We chose 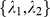, which gives the smallest *BIC* from the candidate models.

### Validation with the synthetic data

We validated the network inference against a set of synthetic data. In the example, shown in Figure S6, the network consists of *k* = 9 nodes with *T* = 4 time-varying states. The transition matrices, 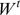 with *t* = 1,2,..,*T*, is designed as sparse *k*-by-*k* matrices with roughly 10% non-zero entries in the range of [−1,+1]. Furthermore, adjacent matrices are designed to be similar with random perturbation of 10 entries in 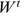 and replacing them in 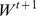 with *t* = 1,2,..,*T*. This is the same policy, used in [42], by collecting samples from each state and adding random noise proportional to the 10% of the signal.

### Comparison with related methods

With respect to network recovery, Ahmed and Xing [42] proposed to recover the network in social and biological studies by a temporally smoothed, 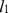-regularized logistic regression formalism. Promising results were reported on reverse engineering of the latent sequence of temporally rewiring political and academic social networks, and the evolving gene networks during the life cycle of Drosophila melanogaster. However, there are several differences between our method and theirs: (i) the loss function in our formulation integrates the error term between the actual and reconstructed gene expression value over multiple time frames in a sliding-window, while in [42] the loss term for each time *t* is the log-likelihood of the model at time *t*; (ii) [42] only considers the closeness between 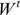
's of directly adjacent time frames, while we can control the clones of all 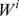
's with a flexible decaying function to adjust the weight that is highly desired in cases of limited training data; (iii) we perform a pre-clustering step to reduce the number of parameters, which also allows us to generate a large number of “pseudo-samples” (data matrices) by sampling from each group. In summary, we have improved the stability of inference by incorporating the pre-clustering step. An alternative approach includes Bayesian techniques, where the notion of sparsity is often enforced by specifying the prior distribution for the graph that panelizes the number of edges. While Bayesian methods have been shown to infer biological networks [43], [44], they (i) are generally greedy in terms of structure inference, and when coupled with advanced MCMC methods to reduce local trapping among possible structures [45], they are compute intensive; (ii) have limitations in terms of inference of feedback loops since network inference is a feed forward action (e.g., network is acyclic) [46]; and (iii) can infer edges based on probabilistic values, and as a result the notion of excitatory and/or inhibitory (e.g., positive and negative edges), they are lost. The main pitfalls are that the number of templates, during the pre-clustering stage, may alter the topology of the causal network, and network inference may be sensitive to a small sample size. The latter is a shortcoming that persists in most systems that infer structures from the data. A potential improvement includes further regularization by incorporating prior knowledge (e.g., KEGG, HPRD) to enforce locality and consistency with the published literature.

## Supporting Information

Figure S1 **Visualization of the consensus matrix of N = 2,3,…,9 clusters for the adaptive dose.**
(TIF)

Figure S2 **Consensus CDF for the consensus matrix of N = 2,3,…,9 clusters for the adaptive dose as shown in Figure S1.**
(TIF)

Figure S3 **Visualization of the consensus matrix of N = 2,3,…,9 clusters for the adaptive dose.**
(TIF)

Figure S4 **Consensus CDF for the consensus matrix of N = 2,3,…,9 clusters for the challenge dose as shown in Figure S3.**
(TIF)

Figure S5 **NUSE (Normalized Unscaled Standard Error) plot of the microarray data.**
(TIF)

Figure S6 **An example of validation data in the top row with positive (red) and negative (green) values in the transition matrices.** The bottom row shows inferred matrices through application of the computational method.
(TIF)

Text S1 **Quality control for microarray data.**
(DOCX)

Text S2 **Selection of the number of clusters from consensus clustering.**
(DOCX)

## References

[1] Barabasi A, Oltvai Z (2004) Network biology: understanding the cell's functional organization. Nature Review Genetics 5: 101–103. 10.1038/nrg1272 14735121

[2] Fauer A, Naldi A, Chaouiya C, Thieffry D (2006) Dynamical analysis of generic Boolean model for control of mammalian cell cycle. Bioinformatics 22: 124–131. 16873462 10.1093/bioinformatics/btl210

[3] McAdams H, Arkin A (1997) Stochastic mechanisms in gene expression. Proceedings of National Academy of Science 94: 814–819. 10.1073/pnas.94.3.814 PMC19596 9023339

[4] Cinquermani E, Millias-Argetitis A, Summers S, Lygeros J (2008) Stochastic dynamics of genetic networks. Bioinformatics 24: 2748–2754. 18845579 10.1093/bioinformatics/btn527

[5] Chaouiya C (2007) Petri Net modeling of biological networks Briefings in Bioinformatics. 8: 210–219. 10.1093/bib/bbm029 17626066

[6] Li S, Brazhnik P, Sobral B, Tyson J (2008) A quantitative study of the division of the cell cycle of Caulobacter Crescentus Stalked cells. PLoS Computational Biology 4. 10.1371/journal.pcbi.0040009 PMC2217572 18225942

[7] Edwards J, Ibarra R, Palsson B (2001) In Silico predictions of Escherichia Coli Metabolic capabilities are consistent with experimental data. Nature Biotechnologyu 19: 125–130. 10.1038/84379 11175725

[8] Karlebach G, Shamir R (2008) Modeling and analysis of gene regulatory networks. Nature Reviews in Molecular and Cell Biology 9: 770–780. 18797474 10.1038/nrm2503

[9] Tenazinha N, Vinga S (2011) A survery of methods for modeling and analyzing integrated biological networks. IEEE Transactions on Computational Biology and Bioinformatics 8: 943–958. 21116043 10.1109/TCBB.2010.117

[10] Tanya K, Hooker A, Zeng G, Sykes P (2007) Low dose X-radiation adaptive response in spleen and prostate of Atm knockout heterozygous mice. International Journal of Radiation Biology 83: 523–534. 17613125 10.1080/09553000701420582

[11] Prise K (2006) New advances in radiation biology. Occupational Medicine 56: 156–161. 16641500 10.1093/occmed/kql010

[12] Tapio S, Jacob V (2007) Radiaoadpative response revisited. Radiation Environment Biophysics 46: 1–12. 10.1007/s00411-006-0078-8 17131131

[13] Nevins J (1988) Toward an understanding of the functional complexity of the E2F and Retinoblastoma families. Cell Growth and Differentiation 9: 585–593. 9716176

[14] Worku D, Jouhra F, Jiang G, Patani N, Newbold R, et al (2008) Evidence of a tumor suppressive function of E2F1 gene in human breast cancer. Anticancer Research 28: 2135–2140. 18751386

[15] Lin W, Lin F, Nevins J (2011) Selective induction of E2F1 in response to DNA damage, mediated by ATM-dependent phosphorylation. Genes and Development 15: 1833–1844. PMC312742 11459832

[16] Barcellos-Hoff M (2005) How tissues respond to damage at the cellular level: Orchestration by transforming growth factor- beta (TGF-β). BJR Suppl 27: 123–127.

[17] Voy B, Scharff J, Perkins A, Saxton A, Borate B, et al (2006) Extracting gene networks for low-dose radiation using graph theoretical algorithms. PLoS Computational Biology 2 7:e89. 16854212 10.1371/journal.pcbi.0020089 PMC1513268

[18] Wichselbaum R, Hallhan D, Fuks Z, Kufe D (1994) Radiation induction of immediate early genes: effectors of the radiation-stress response. Int J Radiation Oncology Biol Phys 30: 229–234. 10.1016/0360-3016(94)90539-8 8083118

[19] Yang J, Xia W, HU M (2006) Ionizing radiation activates expression of FOXO3a, Fas Ligand, Bim, and induces cell apoptosis. International Journal of Oncology 29: 643–648. 16865280 PMC2632978

[20] Thiel G, Cibelli G (2002) Regulation of life and death by the Zinc finger transcription factor Egr-1. Cellular Physiology 193: 287–292. 10.1002/jcp.10178 12384981

[21] Greer E, Brunet A (2008) FOXO transcription factors in ageing and cancer. Acta Physiol 192: 19–28. 10.1111/j.1748-1716.2007.01780.x 18171426

[22] Accili D, Arden K (2004) FoxOs at the crossroads of cellular metabolism, differentiation, and transformation. Cell 117: 421–426. 15137936 10.1016/s0092-8674(04)00452-0

[23] Burgering B, Kpos G (2002) Cell cycle and death control: long live Forkheads. Trends Biochem Sci 27: 352–360. 12114024 10.1016/s0968-0004(02)02113-8

[24] Roy L, Gruel G, Vaurijoux A (2009) Cell responses to ionising radiation analyzed by gene expression patterns. Ann Ist Super Sanita 45: 272–277. 19861732

[25] Turtoi A, Brown I, Oskamp D, Schneeweiss F (2008) Early gene expression in human lymphocytes after gamma irradiation-a genetic pattern with potential biodosimetry. Int J Radiation Biology 84: 375–387. 10.1080/09553000802029886 18464067

[26] Pearl J (2000) Causality: Models, reasoning and inference: Cambridge university Press.

[27] Robin JM, Wasserman L, editors (1999) On the impossibility of of inferring causation from association without background knowledge. Menlo Park, Ca: AAAI Press/MIT Press. 305–321 p.

[28] Das A, Chendil D, Dey S, Mohiuddin M, Mohiuddin M, et al (2001) Ionizing Radiation Down-regulates p53 Protein in Primary Egr-1^−/−^ Mouse Embryonic Fibroblast Cells Causing Enhanced Resistance to Apoptosis. Biological Chemistry 276: 3279–3286. 10.1074/jbc.M008454200 11035041

[29] McBride W, Pajonk F, Chiang C, Sun J (2002) NF-kappa B, cytokines, proteasomes, and low-dose radiation exposure. Mil Med 167: 66–67. 11873521

[30] Pervan M, Iwamoto K, McBride W (2005) Proteasome structure affected by ionizing radiation. Mol Cancer Res 3: 381–390. 16046549 10.1158/1541-7786.MCR-05-0032

[31] Hayflick L (1965) The limited in vitro lifetime of human diploid cell strains. Experimental cell research 37: 614–636. 14315085 10.1016/0014-4827(65)90211-9

[32] Hayflick L, Moorhead P (1961) The serial cultivation of human diploid cell strains. Experimental cell research 25: 585–621. 13905658 10.1016/0014-4827(61)90192-6

[33] Groesser T, Chang H, Fontenay G, Chen J, Costes S, et al (2011) Persistent of gamma-H2AX and 53BP1 fori in proliferating and non-proliferating human mammary epithelial cells after exposure to gamma-rays or iron ions. International Journal of Radiation Research 87: 696–710. 10.3109/09553002.2010.549535 21271785

[34] Han J, Chang H, Yang Q, Fontenay G, Groesser T, et al (2010) Multiscale iterative voting for differential analysis of stress response for 2D and 3D cell culure models. Microscopy 241: 315–326. 10.1111/j.1365-2818.2010.03442.x 21118235

[35] Molller-Levet C, Chu K, Wolkenhauer O (2003) DNA microarray data clustering based on temporal variation: Fcv with tcd preclustering. Bioinoformatics 19: 834–841. 15130832

[36] Ramoni M, Sebastiani P, Kohane P (2002) Cluster analysis of gene expression dynamics. PNAS 99: 9121–9126. 12082179 10.1073/pnas.132656399 PMC123104

[37] Luan Y, Li H (2003) Clustering of time-couse gene expression data using mixed effects model with B-spline. Bioinformatics 19: 474–482. 12611802 10.1093/bioinformatics/btg014

[38] Celeux G, Martin O, Lavergne C (2005) Mixture of linear models for clustering gene expression profiles from repeated microarray experiments. Statistical modeling 5: 243–267.

[39] Monti S, Tamayo P, Mesirov J, Golub TR (2003) Consensus clustering: a resampling-based method for class discovery and visualization of gene expression microarray data. Mach Learn 52: 91–118.

[40] Tibshirani R (1996) Regression shrinkage and selection via the lasso. J Royal Statist Soc B 58: 267–288.

[41] Donoho D (2006) Compressed sensing. IEEE Trans on Information Theory 52: 1289–1306.

[42] Ahmed A, Xing EP (2009) Recovering time-varying networks of dependencies in social and biological studies. PNAS 106: 11878–11883. 19570995 10.1073/pnas.0901910106 PMC2704856

[43] Ellis B, Wong WH (2008) Learning causal Bayesian network structures from experimental data. American Statistical Association 103: 778–789.

[44] Hill SM, Neve RM, Bayani N, Kuo W-L, Ziyad S, et al (2012) Integrated biological knowledge into variable selection: an emprical Bayes approach with an application in cancer biology. BMC Bioinformatics 13: 94. 22578440 10.1186/1471-2105-13-94 PMC3503557

[45] Kou S, Zhou Q, Wong WH (2006) Equi-energy sampler: Applications in statistical inference and satistical mechanics. The annals of Statistics 34: 1581–1619.

[46] Henao R, Winther O (2009) Bayesian sparse factor models and DAGs inference and comparison;. pp. 736–744.

