# Supplementary figures and images for: Inference of Causal Networks from Time-Varying Transcriptome Data via Sparse Coding
Source: PLoS One. 2012 Aug 20;7(8):e42306. doi: 10.1371/journal.pone.0042306 (PMC3423420; doi:10.1371/journal.pone.0042306)

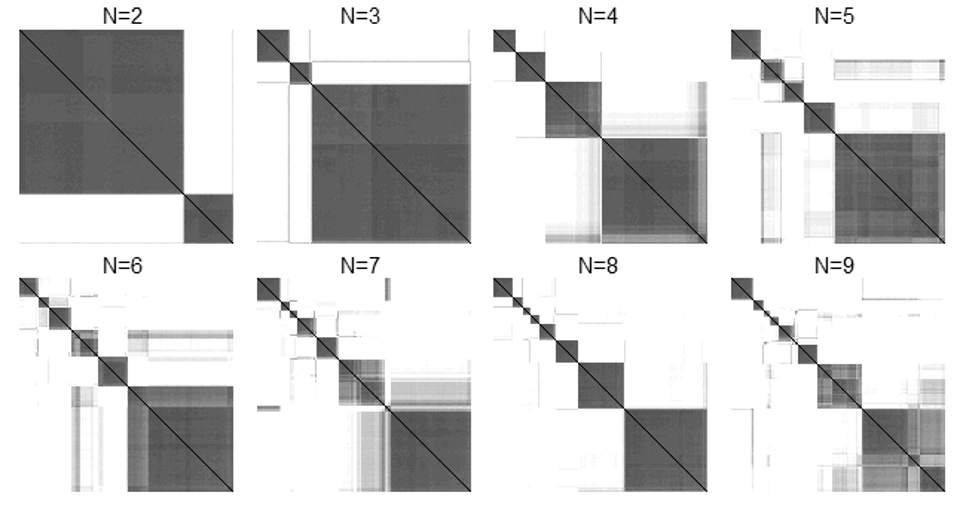

Supplement: Figure S1 — Visualization of the consensus matrix of N = 2,3,…,9 clusters for the adaptive dose. (TIF) [file pone.0042306.s001.tif]

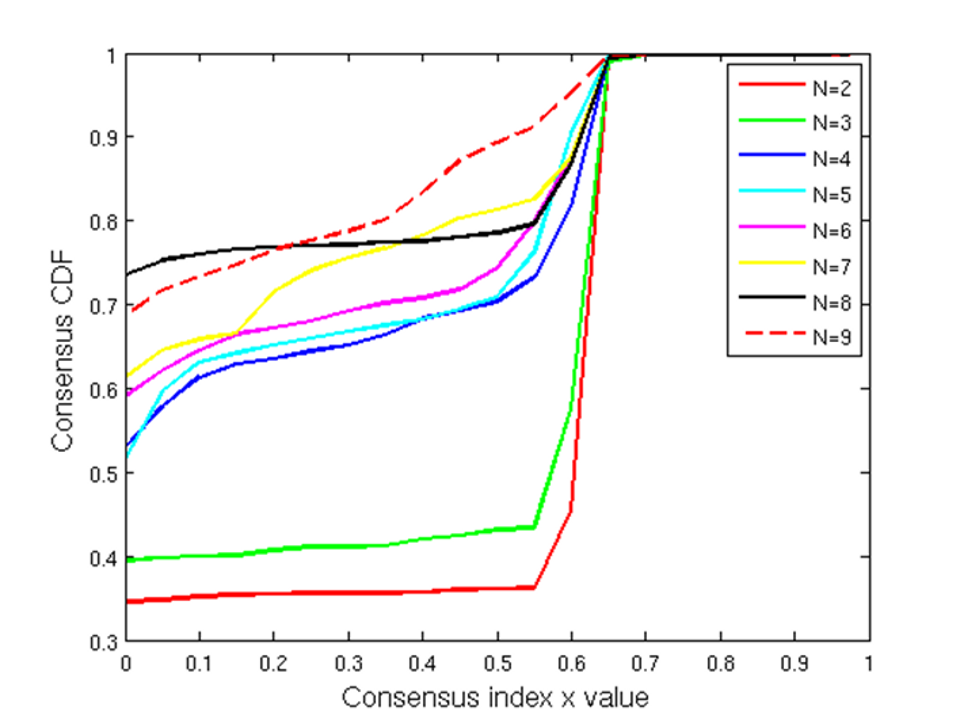

Supplement: Figure S2 — Consensus CDF for the consensus matrix of N = 2,3,…,9 clusters for the adaptive dose as shown in Figure S1. (TIF) [file pone.0042306.s002.tif]

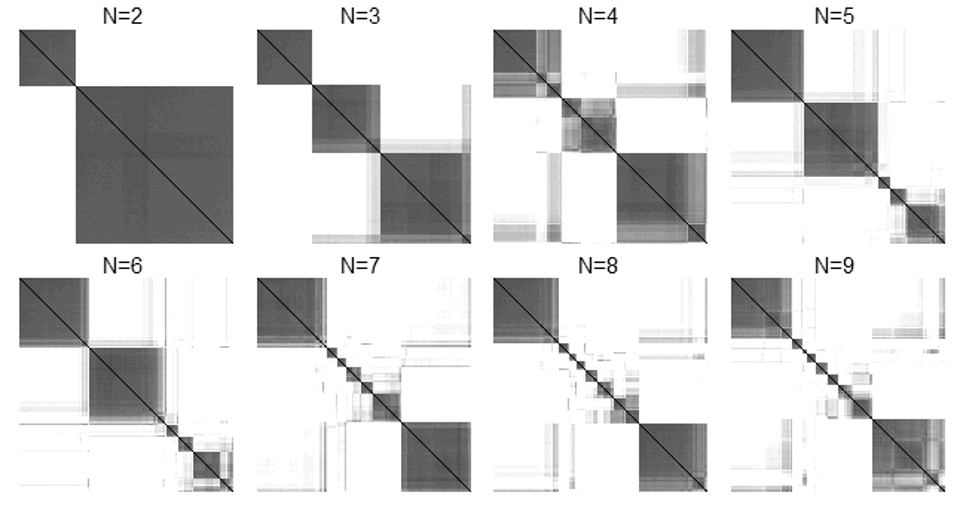

Supplement: Figure S3 — Visualization of the consensus matrix of N = 2,3,…,9 clusters for the adaptive dose. (TIF) [file pone.0042306.s003.tif]

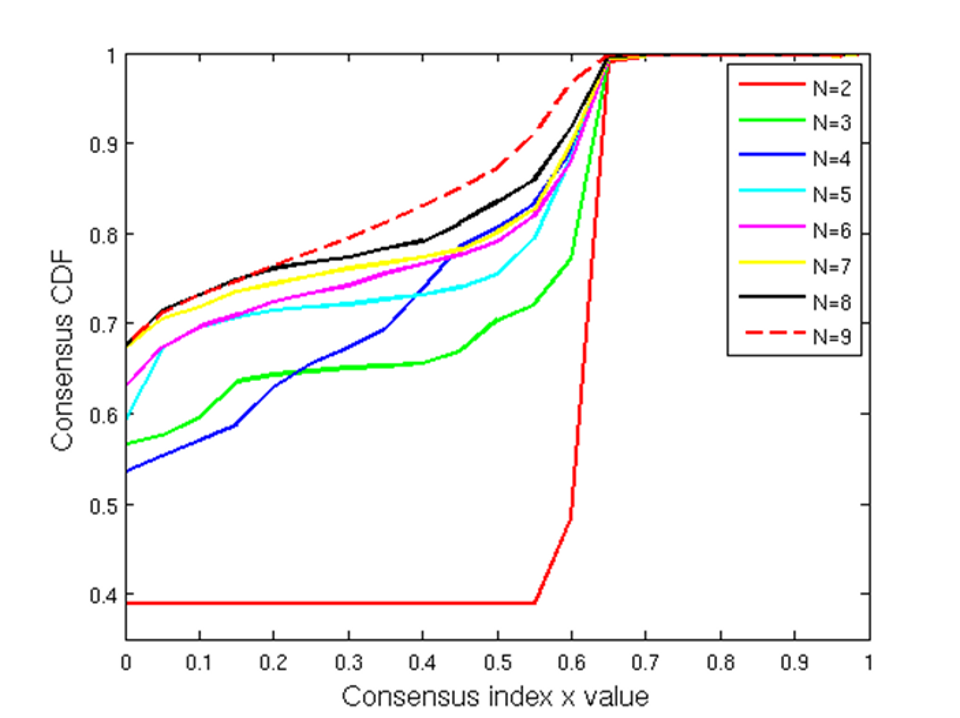

Supplement: Figure S4 — Consensus CDF for the consensus matrix of N = 2,3,…,9 clusters for the challenge dose as shown in Figure S3. (TIF) [file pone.0042306.s004.tif]

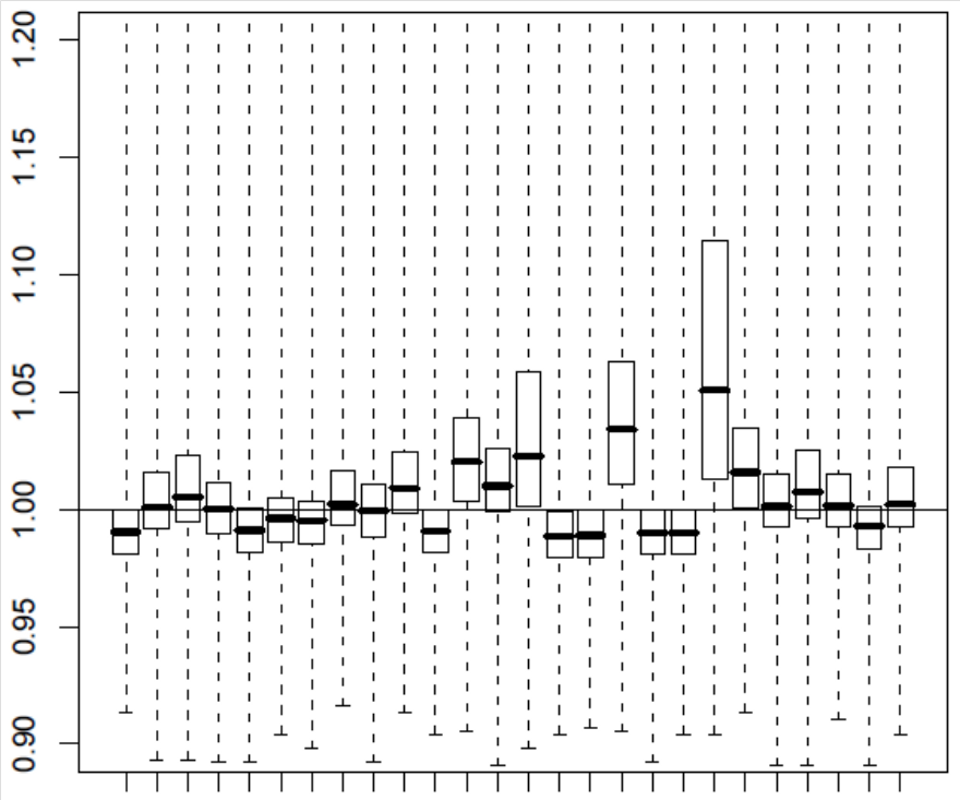

Supplement: Figure S5 — NUSE (Normalized Unscaled Standard Error) plot of the microarray data. (TIF) [file pone.0042306.s005.tif]

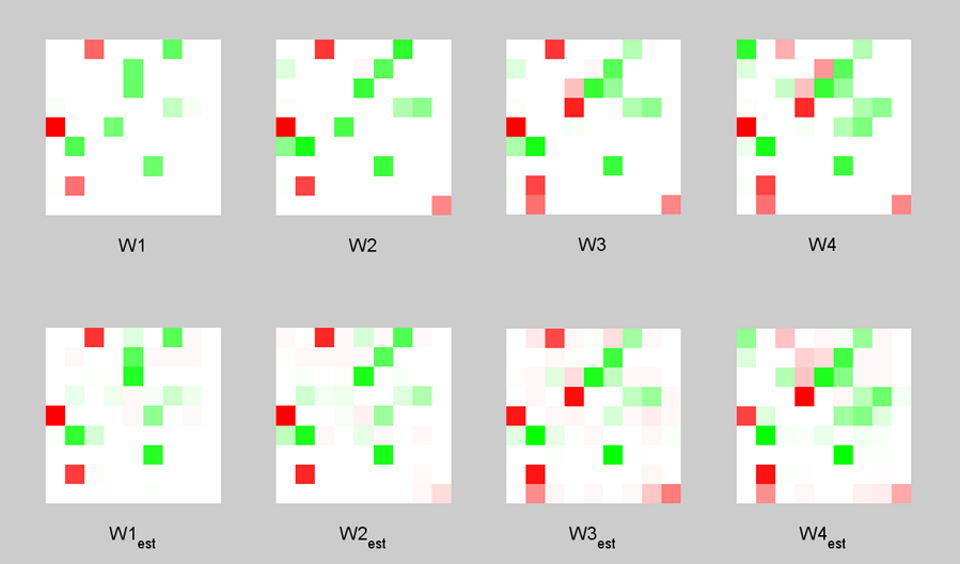

Supplement: Figure S6 — An example of validation data in the top row with positive (red) and negative (green) values in the transition matrices. The bottom row shows inferred matrices through application of the computational method. (TIF) [file pone.0042306.s006.tif]
