# Supplementary material for: Inference of Causal Networks from Time-Varying Transcriptome Data via Sparse Coding
Source: PLoS One. 2012 Aug 20;7(8):e42306. doi: 10.1371/journal.pone.0042306 (PMC3423420; doi:10.1371/journal.pone.0042306)
Supplement: Text S1 — Quality control for microarray data. (DOCX) [file pone.0042306.s007.docx]

Text S1: Quality control for microarray data.

The quality of the microarray data was examined through the Normalized Unscaled Standard Error (NUSE) plot. In a NUSE plot, low quality arrays are significantly elevated or more spread out in comparison with other arrays. The NUSE plot of our microarray data (shown in Figure S5) indicates bad quality for the 20-th array, 0_2Gy_2h_C_(HuGene-1_0-st-v1).CEL, which was excluded from the analysis in the paper.
