# Supplementary material for: Inference of Causal Networks from Time-Varying Transcriptome Data via Sparse Coding
Source: PLoS One. 2012 Aug 20;7(8):e42306. doi: 10.1371/journal.pone.0042306 (PMC3423420; doi:10.1371/journal.pone.0042306)
Supplement: Text S2 — Selection of the number of clusters from consensus clustering. (DOCX) [file pone.0042306.s008.docx]

Text S2: Selection of the number of clusters from consensus clustering.

Consensus clustering of 682 adaptive genes, with four time points, identifies high clustering stability for N=2, 3, and 8 (Figure S1). The consensus CDFs are shown in Figure S2. We chose the eight-cluster result which reveals more details about the class structure. Similarly, consensus clustering of 527 challenge genes, with four time points, identifies high clustering stability for N=2, 3, and 5 (Figure S3). The consensus CDFs are shown in Figure S4. Here, we chose the five-cluster result for the challenge genes.
